# The reality of general surgery training and increased complexity of abdominal wall hernia surgery

**DOI:** 10.1007/s10029-019-02062-z

**Published:** 2019-11-21

**Authors:** F. Köckerling, A. J. Sheen, F. Berrevoet, G. Campanelli, D. Cuccurullo, R. Fortelny, H. Friis-Andersen, J. F. Gillion, J. Gorjanc, D. Kopelman, M. Lopez-Cano, S. Morales-Conde, J. Österberg, W. Reinpold, R. K. J. Simmermacher, M. Smietanski, D. Weyhe, M. P. Simons

**Affiliations:** 1 Department of Surgery and Center for Minimally Invasive Surgery, Academic Teaching Hospital of Charité Medical School, Vivantes Hospital, Neue Bergstrasse 6, 13585 Berlin, Germany; 2 Department of Surgery, Manchester University NHS Foundation Trust, Manchester, M13 9WL UK; 3 Department of General and HPB Surgery and Liver Transplantation, Universitair Ziekenhuis Gent, C. Heymanslaan 10, 9000 Ghent, Belgium; 4 General and Day Surgery Unit, Center of Research and High Specialization for the Pathologies of Abdominal Wall and Surgical Treatment and Repair of Abdominal Hernia, Milano Hernia Center, Instituto Clinico Sant’Ambrogio, University of Insurbria, Milan, Italy; 5 Department of General, Laparoscopic and Robotic Surgery, Chief Week Surgery Departmental Unit, A.O. dei Colli Monaldi Hospital Naples, Naples, Italy; 6 Department of General, Visceral and Oncological Surgery, Wilhelminenspital, 1160 Vienna, Austria; 7 Medical Faculty of Sigmund Freud University, 1020 Vienna, Austria; 8 Surgical Department, Horsens Regional Hospital, Aarhus University, Sundvey 30, 8700 Horsens, Denmark; 9 Unité de Chirurgie Viscérale, Hôpital Privé d’Antony, 1, Rue Velpeau, 92160 Antony, France; 10 Department of Surgery, Krankenhaus der Barmherzigen Brüder, Spitalgasse 26, 9300 St. Veit an der Glan, Austria; 11 Department of Surgery Emek Medical Center, Afula, Israel; 12 Rappaport Faculty of Medicine, Technion, Israel Institute of Technology, Haifa, Israel; 13 Abdominal Wall Surgery Unit, Department of General Surgery, Hospital Universitari Vall d’Hebron, Universitat Autònoma de Barcelona, Passeig Vall d’Hebron 119-129, 08035 Barcelona, Spain; 14 Unit of Innovation in Minimally Invasive Surgery, University Hospital Virgen del Rocío, Av. Manuel Siurot, s/n, 41013 Seville, Spain; 15 Department of Surgery, Mora Hospital, 79285 Mora, Sweden; 16 Wilhelmsburger Krankenhaus Gross-Sand, Gross-Sand 3, 21107 Hamburg, Germany; 17 Department of Surgery, University Medical Center Utrecht, Heidelbergglaan 100, Utrecht, The Netherlands; 18 Department of General Surgery and Hernia Centre, Hospital in Puck, Medical University of Gdansk, Gdansk, Poland; 19 School of Medicine and Health Sciences, University Hospital for Visceral Surgery, Pius Hospital Oldenburg, Medical Campus University of Oldenburg, Georgstr. 12, 26121 Oldenburg, Germany; 20 Department of Surgery, OLVG Hospital, Amsterdam, The Netherlands

**Keywords:** Surgical training, Learning curve, Complex hernias, Tailored approach, Inguinal hernia, Incisional hernia

## Abstract

**Introduction:**

The Accreditation and Certification of Hernia Centers and Surgeons (ACCESS) Group of the European Hernia Society (EHS) recognizes that there is a growing need to train specialist abdominal wall surgeons. The most important and relevant argument for this proposal and statement is the growing acceptance of the increasing complexity of abdominal wall surgery due to newer techniques, more challenging cases and the required ‘tailored’ approach to such surgery. There is now also an increasing public awareness with social media, whereby optimal treatment results are demanded by patients. However, to date the complexity of abdominal wall surgery has not been properly or adequately defined in the current literature.

**Methods:**

A systematic search of the available literature was performed in May 2019 using Medline, PubMed, Scopus, Embase, Springer Link, and the Cochrane Library, with 75 publications identified as relevant. In addition, an analysis of data from the Herniamed Hernia Registry was performed. The percentage of patients with hernia- or patient-related characteristics which unfavorably impacted the outcome of inguinal and incisional hernia repair was also calculated.

**Results:**

All present guidelines for abdominal wall surgery recommend the utilization of a ‘tailored’ approach. This relies on the prerequisite that any surgical technique used has already been mastered, as well as the recognized learning curves for each of the several techniques that can be used for both inguinal hernia (Lichtenstein, TEP, TAPP, Shouldice) and incisional hernia repairs (laparoscopic IPOM, open sublay, open IPOM, open onlay, open or endoscopic component separation technique). Other hernia- and patient-related characteristics that have recognized complexity include emergency surgery, obesity, recurrent hernias, bilateral inguinal hernias, groin hernia in women, scrotal hernias, large defects, high ASA scores, > 80 years of age, increased medical risk factors and previous lower abdominal surgery. The proportion of patients with at least one of these characteristics in the Herniamed Hernia Registry in the case of both inguinal and incisional hernia is noted to be relatively high at around 70%. In general surgery training approximately 50–100 hernia repairs on average are performed by each trainee, with around only 25 laparo-endoscopic procedures.

**Conclusion:**

A tailored approach is now employed and seen more so in hernia surgery and this fact is referred to and highlighted in the contemporaneous hernia guidelines published to date. In addition, with the increasing complexity of abdominal wall surgery, the number of procedures actually performed by trainees is no longer considered adequate to overcome any recognized learning curve. Therefore, to supplement general surgery training young surgeons should be offered a clinical fellowship to obtain an additional qualification as an abdominal wall surgeon and thus improve their clinical and operative experience under supervision in this field. Practicing general surgeons with a special interest in hernia surgery can undertake intensive further training in this area by participating in clinical work shadowing in hernia centers, workshops and congresses.

## Introduction

The Accreditation and Certification of Hernia Centers and Surgeons (ACCESS) Group of the European Hernia Society (EHS) recommends unequivocally a need for the training of specialist hernia surgeons [1]. The most important argument for this recommendation is the increasing complexity of abdominal wall surgery due to new techniques, more difficult cases, a recognized tailored approach and an increasing public awareness which demands nothing short of optimal treatment results [1]. The rising complexity of abdominal wall surgery has led to the international hernia societies publishing several guidelines [2–14]. However, more work is required to define in the scientific literature the growing evolution and complex nature of contemporaneous abdominal wall surgery. Therefore, importance should be given to specific variables contributing to an unfavorable outcome for individual hernias and consequently a clear definition for the more complex procedures in abdominal wall surgery.

The guidelines to date take into account developments in abdominal wall surgery and issue evidence-based recommendations for the best possible contemporary practices in abdominal wall surgery [2–14]. The scope of these guidelines clearly provides information as to how complex abdominal wall surgery has now become and consideration of the myriad of technical details in abdominal wall surgery will inevitably help contribute to the preferred good outcomes [2–14]. Notwithstanding a clinician’s relative experience with the use of a tailored approach for hernia repair, mastery of a single surgical technique is now recognized as possibly insufficient. Therefore, several techniques must now be possibly taught, learned and mastered for each type of hernia to promote a better outcome with minimal morbidity to each individual patient [2–14].

“General surgery training has the intention of training surgeons to a standard for independent practice” [15]. “But there is no worldwide standardization of expected operative experience in general surgery training and various surgical curricula requirements invariably will differ” [15]. “For example, the UK demands 1600 procedures by completion of training and the USA requires 750” [15]. In addition to appendectomy, cholecystectomy and partial colectomy, inguinal/femoral and ventral hernia repairs are listed as part of the core procedures in general surgery training [16].

Based on the available literature, this manuscript aims to first identify factors contributing to the increasing complexity of abdominal wall surgery. Data from the Herniamed Registry and its recognized value are used to determine the proportion of these risk factors applicable to abdominal wall surgery [17, 18]. A collective agreement is then reached to establish whether the requirements for the treatment of the more complex abdominal wall hernias are being met by general surgery training across Europe to then possibly help further, by more objectively enabling surgeons to independently perform these procedures after the appropriate training.

## Materials and methods

A systematic search of the available literature was performed in May 2019 using Medline, PubMed, Scopus, Embase, Springer Link, and the Cochrane Library.

The following search terms were used: “general surgery and training” (2035 hits), “hernia and complex” (1252 hits), “hernia and learning curve” (286 hits), “hernia and tailored approach” (18 hits), “hernia and risk factors” (1306 hits).

**Fig. 1:**
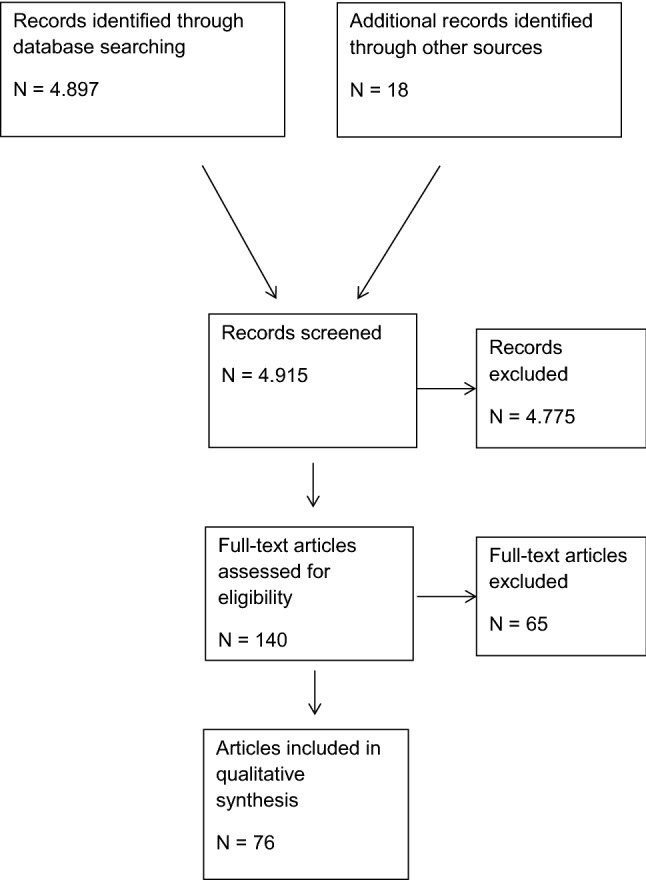
Flow chart of study inclusion

The abstracts of 4915 publications were screened (Fig. 1).

For the present analysis 76 publications were identified as relevant.

Furthermore, an analysis of data from the Herniamed Registry was performed showing the quantitative proportion of the various factors contributing to the complexity of inguinal and incisional hernia.

## Results

### Increasing complexity of abdominal wall surgery

In the literature there are several references which describe the likely unfavorable influences on the outcomes in abdominal wall surgery (Tables 1, 2), thus contributing to the complexity of hernia surgery [19–54]. However, a clear definition of “complex abdominal wall hernia” is still missing and only few attempts have been made to provide such a definition [24]. The majority of factors influencing the complexity of abdominal wall surgery share a common theme for both groin and incisional hernias. Such factors are: Tailored approach, learning curve, emergency setting, obesity (BMI ≥ 30), recurrence, large defects, gender, ASA classification, age ≥ 80 years and risk factors (COPD, diabetes, aortic aneurysm, immunosuppression, cortisone medication, smoking, coagulopathy, anticoagulant or antiplatelet therapy) (Tables 1, 2).

**Table 1:** Factors influencing the complexity of abdominal wall surgery—groin hernia

“Tailored approach”
Learning curve of complex procedures (TEP, TAPP)
Emergency inguinal hernia repair
Obesity (BMI ≥ 30)
Recurrent inguinal hernia
Bilateral inguinal hernia
Groin hernia in women
Scrotal hernia
Inguinal hernia repair and previous lower abdominal surgery
ASA III/IV
Age ≥ 80 years
Risk factors (COPD, diabetes, aortic aneurysm, immune suppression, cortisone medication, smoking, coagulopathy, anticoagulant or antiplatelet medication)

**Table 2:** Factors influencing the complexity of abdominal wall surgery—ventral incisional hernia

“Tailored approach”
Learning curve of complex procedures (Laparoscopic IPOM, open sublay, open component separation technique)
Emergency ventral and incisional hernia repair
Obesity (BMI ≥ 30)
Recurrent ventral and incisional hernias
Large defect size
ASA III/IV
Age ≥ 80 years
Risk factors (COPD, diabetes, aortic aneurysm, immune suppression, cortisone medication, smoking, coagulopathy, anticoagulant or antiplatelet medication)

#### Tailored approach

All guidelines [2–14] for abdominal wall surgery recommend a tailored approach that takes into account the individual patient’s clinical circumstances, diagnosis and preferences. This possibly attests to the fact that no single operative procedure is suitable in all clinical circumstances [2–14]. Consequently, it is perhaps reasonable and understandable that the treating surgeon should at least have adequate experience of not just one surgical technique. It therefore remains unequivocal that any treating surgeon must have already been taught and achieved any learning curve for any surgical techniques recommended in the guidelines. For inguinal hernia surgery a responsible surgeon must therefore be able to undertake proficiently at least the Lichtenstein open mesh procedure as well as a laparo-endoscopic technique, i.e., either the total extraperitoneal patch plasty (TEP) or transabdominal preperitoneal patch plasty (TAPP), and also as non-mesh procedure the Shouldice technique [7].

For the tailored approach in incisional hernia repair the surgeon must learn more techniques, as this repair has many different recognized variations and hence repair choices such as the laparoscopic intraperitoneal onlay mesh (IPOM) technique, open suture, sublay, onlay, IPOM and component separation technique [14]. Another factor requiring expertise is the huge numbers of mesh types with different capacities and indications for use depending on the hernia characteristics. All the above described operative procedures have their own distinctive caveats and require a measured experience and subsequent demonstrable ‘mastery’ or accomplishment of the recognized and accepted learning curve [14]. So any implementation of a tailored approach will require that the responsible surgeon must be experienced in a number of other surgical techniques and is able to demonstrate that they have already ‘mastered’ or reached the desired learning curve. Naturally, this principle can be applied to all the techniques recommended in the guidelines.

Therefore, any implementation of a tailored approach in abdominal wall surgery perhaps confirms the greater complexity that is present but does add to the more stringent demands placed on the surgeon.

#### Learning curve for laparoscopic and open abdominal wall surgery

It is well recognized that laparo-endoscopic surgical techniques are associated with a longer learning curve because of their increased complexity and skill required [19]. For example, the learning curve in the TEP technique for inguinal hernia repair may require up to 250 procedures, especially when taking into account all the outcome criteria [19].

For TAPP the learning curve is seen as approximately 50–100 procedures [19]. Notwithstanding the higher learning curve in TEP and TAPP, the open mesh and non-mesh procedures also have a relevant and defined learning curve [19] which, if not followed correctly, could result in high recurrence and/or chronic pain rates [19]. The laparoscopic IPOM technique in incisional hernia repair is associated with a recognized considerably higher complication rate, especially during the learning curve and therefore there is a strong recommendation that training in this operation is always under close supervision [19]. At present there are unfortunately no data available as such on the expected or recommended learning curve for the open surgical techniques used for incisional hernia repair [19]. But it can be assumed that the open techniques, such as the sublay operation, open IPOM and the component separation techniques, are associated with significantly longer learning curves than inguinal hernia repairs. Hence, implementation of any ‘tailored’ approach with ‘mastery’ and expertise developed in many operative procedures in abdominal wall surgery to come in line with the current guidelines will invariably significantly add to the already increasing demands on any surgeon. Appropriate training in line with the guidelines will generally rely on extensive training under the supervision of an experienced abdominal wall surgeon rather than on an ad hoc approach which can be assumed to be commonly employed in most general surgical training environments [19].

#### Emergency abdominal wall repair

Emergency hernia repair is associated with an increased risk of morbidity and mortality [20]. In a study from the USA the mean mortality rate was 3.3% [20]. There is a noted increase in incidence of emergency hernia procedures over 10 years from 16.0 per 100,000 person-years in 2001 to 19.2 per 100,000 person-years in 2010 [20]. This increased incidence per 100,000 person-years was seen, particularly, amongst men aged ≥ 65 years of age (2001:50.1–2010:71.3).

From the registry data, the proportion of emergency procedures in the Swedish Hernia Registry for inguinal hernia was 5.1% and for femoral hernia 36.5% [19]. In the Danish Hernia Database the proportion of emergency procedures for groin hernia was 3.6% [20]. The proportion of emergency incisional hernia procedures in the Herniamed Registry was 3.1% [23].

#### Obesity

Obesity is one of the recognized important criteria when considering defining a complex abdominal wall hernia [24]. “Obesity poses specific risks as well as challenges to surgeons who deal with abdominal wall reconstruction” [25]. In an analysis of the American College of Surgeons National Surgical Quality Improvement Program (ACS-NSQIP) 61.4% of 55,180 patients with minimally invasive ventral hernia repair had a body mass index (BMI) > 30 kg/m^2^ [26]. Increased incidence of surgical and medical complications is significantly associated with a high BMI (*p* < 0.0001) [26].

In a cohort of 102,191 patients with open ventral hernia repair, 58.5% were obese [27]. When stratified by body mass index class, higher classes were associated with an increase in all postoperative complications (*p* < 0.0001), demonstrating a direct link with a steady increase in complication rates with increasing body mass index [27].

In a clinical outcomes’ analysis comparing laparoscopic versus open inguinal hernia repair of the American College of Surgeons National Surgical Quality Improvement Program (ASC-NSQIP) 7346 from 46,793 (16.3%) patients were obese with a BMI ≥ 30 kg/m^2^ [26].

#### Recurrent hernias

In registry analyses for both the laparo-endoscopic and open techniques a more unfavorable outcome has been identified for recurrent inguinal hernia compared with primary inguinal hernia repairs [29, 30]. Accordingly, there is great emphasis placed on the fact that recurrent inguinal hernia repairs should be managed by a more expert surgeon [29, 30]. The inguinal hernia recurrence rate in the ‘total collective’ of inguinal hernias is 11–13% [29–31]. As per the guidelines’ recommendation, recurrent inguinal hernia after prior anterior repair should be treated with a laparo-endoscopic technique and recurrence after a posterior repair technique with an open anterior technique but always depending upon the expertise of the individual surgeon [7].

Recurrent incisional hernia following primary mesh procedure is now classified and regarded as a complex abdominal wall hernia [24]. The proportion of recurrent incisional hernias in the total collective of incisional hernias is moderately high at 22% [32]. There are though very few studies on the treatment and outcome for recurrent incisional hernia to date [32]. In conclusion, any recurrent procedures undertaken for abdominal wall reconstruction, when there is already a mesh implant in the abdominal wall, reasonably requires a highly experienced surgeon [32] and a laparo-endoscopic approach for such a recurrence is recognized as especially demanding.

#### Bilateral inguinal hernias

In the international guidelines for groin hernia management of the HerniaSurge Group laparo-endoscopic repair is recommended for the repair of primary bilateral inguinal hernias [7]. The rate of bilateral inguinal hernias in laparo-endoscopic approach from registry analyses is 28% [33, 34]. The postoperative complication rates following bilateral inguinal hernia repair are known to be significantly higher than after unilateral inguinal hernia repairs [33, 34]. Accordingly, bilateral inguinal hernia repair using a laparo-endoscopic technique recommends the use of an appropriately experienced and trained surgeon [33, 34].

#### Groin hernia in women

The proportion of women in the total collective of inguinal hernias is 8.0–11.5% [35], with 16.7–37% of women found to have femoral hernias [35]. The rate of emergency procedures at 14.5–17.0% is 3–4 times higher than in men [35]. Therefore, ‘watchful waiting’ is not indicated for women with a diagnosis of a groin hernia [35]. The guidelines recommend a laparo-endoscopic technique for repair of femoral hernia in women because of its superior diagnostic and therapeutic value [7]. In systematic reviews and registry analyses, women have been found to be at increased risk for developing chronic pain following repair [36, 37]. As such, groin hernia repair in women has more caveats and places an added ‘stringent’ demand on the surgeon [37].

#### Scrotal hernias

In the guidelines of the European Association of Endoscopic Surgery (EAES), scrotal hernias are classified as being a complex condition [6, 38]. The challenge in scrotal hernia repair is to ensure complete dissection of the large hernia sac from the inguinal canal and scrotum [4, 5, 38]. Failure to remove a large part of the hernia sac will generally result in a persistent seroma [4, 5, 38]. “Endoscopic control of bleeding during scrotal hernia repair often is also recognized as being more difficult, especially when dissecting the hernia sac from the spermatic cord structures” [4, 5, 38]. “Therefore, there is a higher incidence of postoperative secondary hemorrhage and hematoma formation” in such hernias [4, 5, 38]. “The EHS guidelines therefore recommend the open mesh technique as the procedure of choice for a large scrotal hernia” [2, 3, 38].

“HerniaSurge Group does though suggest individualization (open or TAPP) in large scrotal or irreducible hernia” and in so takes into account the relative expertise of the surgeon involved [7, 38].

In the registry data available the proportion of scrotal hernia repairs is 2% for TEP and 3% for TAPP [39] and the proportion of scrotal hernias in the total patient collective of inguinal hernias is 6% [40].

#### Large defects in ventral and incisional hernia repair

Large and giant ventral incisional hernia repair carry a recognized higher risk of postoperative complications [41].

The proportion of incisional hernias with a defect of ≥ 10 cm in the vertical or horizontal dimension is stated as 15% [41].

Large defect sizes have a recognized negative influence on the perioperative complication rates and consequently the long-term outcomes [42, 43]. Increased hernia size leads to an increased risk of complications [44].

#### ASA score

In a systematic review of the perioperative complications of inguinal hernia repair, a high ASA score had an unfavorable influence on the outcome [45].

This fact is also confirmed by registry analysis for both TEP and the Lichtenstein repair [46]. Likewise, for ventral incisional hernias the negative influence of a high ASA score on the rate of surgical site infections was also demonstrated [47]. In a multivariable analysis of 5214 laparoscopic intraperitoneal onlay mesh repairs of incisional hernias, an ASA score of III/IV versus a score of I demonstrated a significantly higher risk for the development of a recurrence [43].

#### Age > 80 years

Examining endoscopic inguinal hernia surgery the rate of perioperative complications increases in octogenarians and above [48]. In the Spanish National Registry of Incisional Hernia (EVEREG) increasing incisional hernia complication rates was observed from age > 70 years [49]. Therefore, it is very reasonable that the indication for surgery of an incisional hernia in an older patient should be carefully and critically considered [50].

#### Risk factors

Other potential risk factors for an unfavorable outcome in hernia surgery are chronic obstructive pulmonary disease, diabetes, aortic aneurysm, immunosuppression, corticosteroid treatment, smoking, coagulopathy, antiplatelet medication and anticoagulation therapy [44, 46, 51–53].

In inguinal hernia these risk factors have a recognized negative influence on the postoperative complication rates as well as the complication-related reoperation rates [46]. Likewise, in incisional hernia repair the negative impact of risk factors on the postoperative complication rates have also been clearly demonstrated [53].

#### Previous lower abdominal surgery

In a series of 301 inguinal hernia repairs, 105 patients (34.9%) had previously undergone lower abdominal surgery [54]. In complex scenarios the guidelines recommend that only a very experienced laparo-endoscopic hernia surgeon should perform a minimally invasive procedure [4–6]. In the new international guidelines the HerniaSurge Group recommend the use of the open Lichtenstein technique in this circumstance [7].

### Proportion of more complex inguinal and incisional hernias in the total collective

To date, no data have been published showing the proportion of patients with inguinal or incisional hernia exhibiting one or more characteristics of a complex hernia. Therefore, an analysis of data from the Herniamed Registry was performed and the findings are presented below.

In the last data analysis up to February 1, 2019, of the total 612,830 prospectively documented cases in the Herniamed Registry, there were 401,446 inguinal hernias in the database. There were 394,088 patients with complete data entry and 392,035 with an age of ≥ 16 years. The proportion of emergency inguinal hernia procedures was *n* = 10,350 (2.64%). 46,720 (11.92%) of patients had an inguinal hernia recurrence. 69,200 (17.65%) had undergone bilateral repair. The proportion of women was *n* = 46.369 (11.83%). 13,166 (3.36%) cases were classified as scrotal hernia. 60,613 (17.76%) had undergone previous surgery of the lower abdomen.

41,501 (10.63%) of patients had a BMI ≥ 30 kg/ m^2^. 64,102 (16.35%) patients with inguinal hernia had been classified as ASA III/IV. The number of patients > 80 years of age was *n* = 27,961 (7.13%). At least *n* = 27,961 (7.13%) patients possessed one recorded risk factor (chronic obstructive pulmonary disease, diabetes, aortic aneurysm, immunosuppression, corticosteroid treatment, smoking, coagulopathy, antiplatelet medication and anticoagulation therapy).

On summation of all the characteristics and factors related to inguinal hernia repair that demonstrated an unfavorable influence on the outcome, *n* = 280,593 (71.57%) patients had a recorded characteristic and/or factor. This resulted in *n* = 111,442 (28.43%) patients who were not at high risk of more negative outcomes. These cases represented elective, primary, unilateral, non-scrotal inguinal hernias in men who had no other risk factors.

Examining the number of influencing factors leading to a more negative outcome, *n* = 136,444 (34.80%) patients had one factor, *n* = 85,482 (21.80%) two, *n* = 40,160 (10.24%) three, *n* = 14,260 (3.64%) four, *n* = 3657 (0.93%) five, *n* = 553 (0.14%) six, *n* = 36 (0.001) seven and *n* = 1 (0.00%) eight.

Of the 612,830 patients in the Herniamed Registry database, 70,748 had a defined incisional hernia. Of these, 68,923 had a complete data set and 68,812 an age of ≥ 16 years.

For incisional hernia the proportion of emergency procedures was *n* = 3582 (5.21%) and the proportion of incisional hernia recurrence *n* = 14.482 (21.05%). The proportion of patients with a hernia defect width of > 10 cm (European Hernia Society classification W3) [24] was *n* = 11,809 (17.16%). The number of patients with ASA score III/IV was *n* = 23,179 (33.68%).

The number of patients aged > 80 years was *n* = 4660 (6.77%). In the incisional hernia collective group *n* = 28,787 (41.83%) patients had at least one risk factor (chronic obstructive pulmonary disease, diabetes, aortic aneurysm, immunosuppression, corticosteroid treatment, smoking, coagulopathy, antiplatelet medication and anticoagulation therapy).

On summation of all the factors and characteristics related to incisional hernia repair that increased the risk of a negative outcome applied to *n* = 48,722 (70.80%) of patients. Therefore, only *n* = 20,090 (29.20%) of cases with incisional hernia were not at an increased predisposition of possible negative outcome. These related to elective, primary, small to medium-sized incisional hernias in patients with no other risk factors.

On summation of the complex influencing factors related to incisional hernia repair, *n* = 22,582 (32.82%) had one factor, *n* = 16,767 (24.37%) two factors, *n* = 7343 (10.67%) three factors, *n* = 1810 (2.63%) four factors, *n* = 206 (0.30%) five factors with *n* = 14 (0.02%) six factors.

In summary, approximately 70% of all patients with inguinal and incisional hernia had negative influencing factors on the outcome. Of these 70% of patients, in turn around 36% with inguinal hernia and around 38% with incisional hernia had several factors that exerted a possible negative effect on the outcome.

As the Herniamed database is voluntary and only covers around 20% of German hernia patients there is a possible inclusion bias. Many dedicated German hernia surgeons include their patients, probably involving a higher rate of complex cases that have been referred to them. Relatively large numbers of easy cases are possibly treated by surgeons not enrolled in the database. Their results are unknown. Despite this bias the Herniamed database has huge power and relevance in this study. It is the largest database that includes all the relevant risk factors and patient characteristics.

### General surgery training and hernia repair

“The American Board of Surgery has designated 132 procedures as being core to the practice of general surgery” [16]. “General surgery residents are expected to be able to safely and independently perform those designated procedures by the time they graduate/are board certified” [16]. “There is increasing concern though that perhaps some general surgery residents are not competent to enter independent practice” [16]. In a study from a US university hospital approximately 40% of the faculty members expressed that trainees were not independently capable of performing inguinal hernia repair at any stage of their training [55]. This is reflected in the fact that “US General Surgery residents are reported to be not universally ready to independently perform core procedures (appendectomy, inguinal hernia repair, cholecystectomy) by the time they complete residency training” [16]. “Eighty percent of US general surgery residents do undertake a period of post-residency fellowship training which is mirrored with 77% of general surgery trainees in the UK also pursuing additional clinical fellowship periods, in addition to their standard specified training” [56, 57]. “This comprehensively suggests that the majority of trainees in both countries do feel the need to extend their clinical training before independent practice” [56, 57].

What is apparent in contemporary training was demonstrated by a meta-analysis of 12 studies that reported the actual numbers of inguinal hernia repairs performed by general surgery trainees [15]. Two of these studies were from the UK, one from Thailand and nine from the USA [58–69]. The mean figure reported for general surgery training in the USA was 53–71 inguinal hernia repairs [15].

One US study did report a greater number of repairs with a mean of 113 hernia repairs per trainee [15]. UK studies overall reported a mean of 90 inguinal hernia repairs by the residents during their general surgery training.

A further study from the UK, which was published after the meta-analysis, reported a mean of 117 repairs for the index procedure inguinal hernia repair for 311 trainees [56]. By contrast, 69 trainees from a single UK Deanery had performed only a mean of 64 inguinal hernia repairs [70].

“Guidance for the award of a certificate of completion of training in the UK also stipulates that, among the competences defined in the general surgery curriculum, trainees should be able to demonstrate that they have performed a minimum number of logged surgical procedures” [71]. This minimum number of operative procedures for inguinal hernia was given as 60 procedures [71]. But no further information is provided for ventral incisional hernia repair procedures [71]. In Germany there is a requirement for evidence to be provided of having conducted at least 50 hernia repairs during 6 years of general surgery training [72].

In Switzerland, general surgery trainees need to have performed at least 40 inguinal or umbilical hernia repairs and 25 abdominal wall hernia repairs over a 6-year period [73].

Only very few studies have calculated what proportion of the total number of procedures performed during general surgery training were laparo-endoscopic inguinal hernia repairs. For a mean total number of 71.2 inguinal hernia repairs undertaken by graduating general surgery residents in 2010/2011, McCoy et al. [60] reported a mean of 23.3 repairs using a laparo-endoscopic technique. For a mean total number of 67.4 inguinal hernia repairs conducted by graduating surgical residents in 2010, Unawane et al. [61] reported a mean of 20.4 repairs using again a minimally invasive technique. Fryer et al. [62] reported on the number of procedures undertaken by 15 general surgery program graduates of a single institution, with on average a higher than previously reported numbers; 92.0 open inguinal hernia repairs with the general surgery program graduates performing a mean number of 21.1 laparo-endoscopic inguinal hernia repairs. Carson et al. [64] analyzed the number of individual cases performed by all graduating chief residents from all general surgery residency programs in the United States. Of the total mean number of 62,462 inguinal hernia repairs performed in the years 2007/2008, 25.8% were conducted using a laparo-endoscopic technique. This value corresponds to a mean number of 16 minimally invasive procedures. Bell et al. [65] reported on 1022 US general surgery residents who graduated in 2005 and who, in addition to an average of 45.9 open inguinal hernia repairs, performed 12.7 by a laparo-endoscopic technique. In a retrospective review of the Accreditation Counsel for Graduate Medical Education the average number of laparo-endoscopic inguinal hernia repairs performed by graduating surgical residents was not greater than 34.1 during the period of 2015–2018 [75]. There is though disappointingly a noted paucity of data available in the literature on the role of ventral and incisional hernia repair in general surgery training, Malangoni et al. [59] reported on 1923 residents who completed their general surgery training in 2010–2011 with the residents performing on average 43.5 ventral hernia repairs during their training. Fryer et al. [62] did report a mean number of 48.9 ventral hernia repairs for 15 residents in a single institution during 5 years of general surgery training, which was not too dissimilar to other findings.

## Conclusions

The analysis of the current literature coupled with data from the Herniamed Hernia Registry provides an insight into the complexity of abdominal wall surgery and where training of such operations stands to date. It can be reemphasized that the use of a tailored approach to inguinal and incisional hernia surgical repair perhaps is now making more tightened demands on the surgeon since a plethora of defined surgical techniques are now recognized, with each having their own learning curves. These techniques must consequently be firstly taught and then mastered. Based on the guidelines [2–14], implementation of such a tailored approach is a prerequisite now for low-risk and effective abdominal wall surgery.

Notwithstanding the tailored approach, there are also several factors that have a recognized unfavorable influence on the outcome of hernia surgery, thus placing a greater effect on the caveat of complex abdominal wall surgery. The likely unfavorable factors include the following hernia- and patient-related characteristics:

emergency operation, obesity, recurrent hernia, bilateral inguinal hernia, groin hernia in women, scrotal hernia, defect size, ASA score, age > 80 years, medical risk factors and previous lower abdominal surgery.

These factors which may adversely affect the outcomes are unfortunately not rare and seen in at least 70% of all patients, as demonstrated by data in the Herniamed Hernia Registry on inguinal and incisional hernia. Almost 35% of all inguinal and incisional hernia patients possess more than one factor which increases the morbidity of patients undergoing a hernia repair and so negatively influences the outcome of the repair. So in essence considering the need for the tailored approach, the required steep learning curves for the individual surgical techniques and the high incidence of co-morbid factors, the complexity of abdominal wall surgery becomes apparent. A simple and relevant question can then be posed as to whether general surgery training in real terms is meeting the requirements for adequate training in abdominal wall surgery as set out by the guidelines? Fifty to 100 procedures with a proportion of 25 laparo-endoscopic repairs appears inadequate to technically overcome any learning curve associated with a specified surgical technique (TEP or TAPP, Lichtenstein, Shouldice, laparoscopic IPOM, open sublay, open or endoscopic component separation technique, open onlay) [19]. Therefore, the ACCESS Group advises that all general surgeons should be better trained in order to become proficient in abdominal wall surgery by first of all overcoming the learning curve of both open and laparo-endoscopic hernia procedures as recommended by the current guidelines with direct and guided supervision [1].

“Fellowship posts after general surgery training are increasingly common and offer targeted opportunities for training and personal development” [74]. In a recent survey, over three-quarters of trainees have or will have undertaken a clinical fellowship after completion of their general surgical training [74]. “Competence, confidence and subspecialty skill development are the core main aims” [74].

It is well recognized and also commended that young surgeons show widespread willingness to consolidate their clinical and operative experience under supervision by undertaking clinical fellowships after core general surgery training.

Therefore, it is entirely reasonable that a program should be developed to allow and facilitate young general surgeons wishing to expand their knowledge and experience in abdominal wall surgery. But practicing general surgeons with a special interest in hernia surgery can also extend their knowledge by participating in clinical work shadowing in hernia centers, workshops and congresses.

This need for abdominal wall surgery training with its complexity, varied techniques and dealing with patients with on average higher comorbidities has been demonstrated by the data above.

In summary, the Access Group recommends the implementation for a recognized and credited specialist in abdominal wall surgery. As experience in hernia surgery is difficult to measure [76], patient characteristics and outcomes should be followed by a registry [1].
